# True Katydids (Pseudophyllinae) from Guadeloupe: Acoustic Signals and Functional Considerations of Song Production

**DOI:** 10.1673/031.013.15701

**Published:** 2013-12-24

**Authors:** Andreas Stumpner, Angela Dann, Matthias Schink, Silvia Gubert, Sylvain Hugel

**Affiliations:** 1 Georg-August-University of Göttingen, Dept. of Cellular Neurobiology, JFB-Institute of Zoology and Anthropology, Schwann-Schleiden-Centre for molecular cell biology, Julia-Lermontowa-Weg 3, 37077 Göttingen, Germany; 2 Present address: Institut für Experimentelle Pneumologie, Max-Lebsche-Platz 31, 81377 München; 3 Present address: Georg-August-University of Göttingen, Dept. of Systemic Neurobiology, JFB-Institute of Zoology and Anthropology, von-Siebold-Str. 4, 37073 Göttingen, Germany; 4 INCI, CNRS, Université de Strasbourg, Strasbourg, France

**Keywords:** bioacoustics, bush-cricket, stridulation

## Abstract

Guadeloupe, the largest of the Leeward Islands, harbors three species of Pseudophyllinae (Orthoptera: Tettigoniidae) belonging to distinct tribes. This study examined the basic aspects of sound production and acousto-vibratory behavior of these species. As the songs of many Pseudophyllinae are complex and peak at high frequencies, they require high quality recordings. Wild specimens were therefore recorded ex situ. Collected specimens were used in structure-function experiments. *Karukerana aguilari* Bonfils (Pterophyllini) is a large species with a mirror in each tegmen and conspicuous folds over the mirror. It sings 4–6 syllables, each comprising 10–20 pulses, with several peaks in the frequency spectrum between 4 and 20 kHz. The song is among the loudest in Orthoptera (> 125 dB SPL in 10 cm distance). The folds are protective and have no function in song production. Both mirrors may work independently in sound radiation. *Nesonotus reticulatus* (Fabricius) (Cocconotini) produces verses from two syllables at irregular intervals. The song peaks around 20 kHz. While singing, the males often produce a tremulation signal with the abdomen at about 8–10 Hz. To our knowledge, it is the first record of simultaneous calling song and tremulation in Orthoptera. Other males reply to the tremulation with their own tremulation. *Xerophyllopteryx fumosa* (Brunner von Wattenwyl) (Pleminiini) is a large, bark-like species, producing a syllable of around 20 pulses. The syllables are produced with irregular rhythms (often two with shorter intervals). The song peaks around 2–3 kHz and 10 kHz. The hind wings are relatively thick and are held between the half opened tegmina during singing. Removal of the hind wings reduces song intensity by about 5 dB, especially of the low frequency component, suggesting that the hind wings have a role in amplifying the song.

## Résumé

La Guadeloupe, la plus grande des Iles Sous le Vent, abrite trois espèces de Pseudophyllinae (Orthoptera : Tettigonidae) appartenant à des tribus distinctes. Notre objectif était d'étudier les aspects basiques de la production de son et le comportement acoustique/vibratoire de ces espèces. Ces objectifs requièrent des enregistrements de qualité élevée car la stridulation de nombreux Pseudophyllinae est complexe et de haute fréquence. Nous avons donc enregistré des spécimens sauvages ex situ. Nous avons profité de la disponibilité de ces spécimens en laboratoire pour étudier la relation structure-fonction de leur système stridulatoire. *Karukerana aguilari* Bonfils (Pterophyllini) est une grande espèce possédant sur chaque tegmen un miroir recouvert par un large repli. Son chant comprend 4–6 syllabes, comprenant chacune 10–20 pulses, avec plusieurs pics dans le spectre de fréquence entre 4 et 20 kHz. Il s'agit de l'une des stridulations d'orthoptères les plus fortes (> 125 dB SPL à 10 cm de distance). Les replis ont une fonction protectrice et ne sont pas impliqués dans la stridulation. Les deux miroirs semblent impliqués de manière indépendante dans la production du son. *Nesonotus reticulatus* (Fabricius) (Cocconotini) produit des phrases (« verses ») de deux syllabes à intervalles irréguliers. Le maximum de fréquence est d'environ 20 kHz. En stridulant, les mâles produisent souvent une trémulation avec l'abdomen à environ 8 – 10 Hz. A nôtre connaissance, il s'agit du premier cas documenté de trémulation produite en même temps que le chant d'appel chez les orthoptères. Les autres mâles répondent à la trémulation en trémulant à leur tour. *Xerophyllopteryx fumosa* (Brunner von Wattenwyl) (Pleminiini) est une grande espèce mimétique d'écorce et produisant des syllabes de environ 20 pulses. Les syllabes sont produites à un rythme irrégulier (souvent par deux avec un intervalle court). Les maxima de fréquence sont d'environ 2–3 kHz et 10 kHz et évoquent un coassement. Durant la stridulation, les ailes postérieures qui sont relativement épaisses sont maintenues entre les élytres entre-ouverts. L'ablation des ailes postérieures réduit l'intensité de la stridulation d'environ 5 dB, particulièrement dans les basses fréquences du spectre, suggérant un rôle des ailes postérieures dans l'amplification de la stridulation.

## Introduction

Bush-crickets, also known as katydids (Orthoptera: Tettigoniidae), are a highly diverse group in the subordera Ensifera and occur in a variety of habitats around the world, from extremely dry to tropical rainforest (e.g., Bailey and Rentz 1998; Gwynne 2001). With few exceptions, bush-crickets produce songs with the main function of mate attraction (Heller 1988; Gwynne 2001; Korsunovskaya 2008; Monteallegre 2009). Most bush-crickets produce sounds by stridulation, i.e., by rubbing their tegmina against each other. Among subfamily Pseudophyllinae, a variety of specializations have developed, such as extremely low or extremely high song carrier frequencies and tremulation signals as additional cues for communication (Morris et al. 1994; Heller 1995; Römer et al. 2010). Stridulation and tremulation allow for varying the communication distance and may be produced depending on predatory pressures (Römer et al. 2010). All in all, relatively few detailed descriptions of songs and the production of songs are published (e.g., Monteallegre et al. 2003). When observing behavior of three Pseudophyllinae from Guadeloupe Island, species specific aspects of communication regarding song structure, usage of signals other than acoustic ones, and undescribed aspects of song production were noted in all three species. Therefore, this study aimed to describe the basic elements of the communication system of all three species, none of which has been published before, with a focus on the specific peculiarities of the singing apparatus or song production in each of the species. As recordings were preformed ex situ on wild specimens carried from Guadeloupe to the laboratory, structure/function experiments were also performed on these specimens.

## Materials and Methods

### Location

Guadeloupe (16° 15′ N, 61° 35′ W) is the largest of the Leeward Islands in the Lesser Antilles. It consists of two islands separated by a narrow channel. The eastern island is of karstic origin, with hills and plains; it is mostly cultivated but harbors dry and semi-dry forests. The western island is of volcanic origin; it is more preserved and harbors a low-land forest and a mountain rain forest. These latter are part of Parc National de la Guade-loupe.

### Species

**Figure 1.**
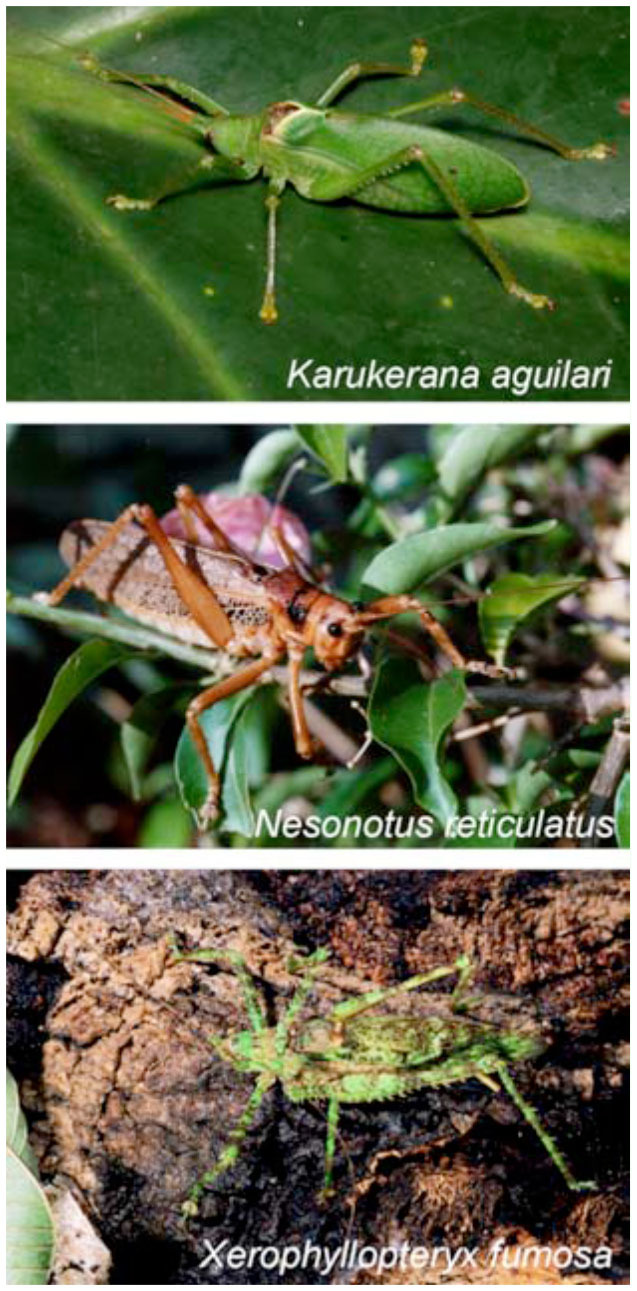
Photos of three Pseudophyllinae from Guadeloupe taken in the field (S. Hugel). High quality figures are available online.

The following three species of Pseudophyllina were collected in the field (authorization from Office National des Forêts and the Parc National de la Guadeloupe): *Karnkerana aguilari* Bonfils, 1965 (Pterophyllini), *Nesonotus reticulatus* (Fabricius, 1793) (Cocconotini), and *Xerophyllopteryx fumosa* (Brunner von Wattenwyl, 1895) (Pleminiini) (Figure 1). All specimens were collected in summer 2004 in Guadeloupe, while one individual of *X. fumosa* was caught in Guadeloupe in 2007. *N. reticulatus* was held in a low density laboratory culture at around 27° C and high humidity from 2004 on. The behavior of nine male and two female *N. reticulatus* was studied, as well as the stridulatory apparatus of three males. Four male *K. aguilari* were available for morphology and one for recordings, while five individuals of *X. fumosa* were studied for morphology, and two were used for sound recordings.

### Scanning electron microscopy

The scanning electron microscope used was either a Jeol JSM-6360 (www.jeol.com) in low vacuum mode with an acceleration of 18 MeV, or a Philips XL-30 ESEM (www.philips.com) with an acceleration of 20 MeV. Specimens were directly imaged with-out metallization. Scanning electron microscopy pictures were taken of one repre-sentative specimen.

**Video 1.**
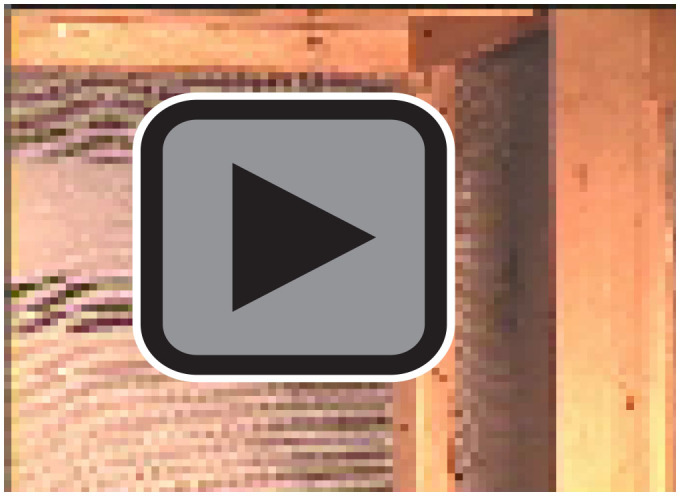
Recording of an intact *Xerophyllopteryx fumosa* male in a cage. The bright spot on the left tegmen represents a piece of reflecting foil for recording wing movements. Download video

### Recording of songs and vibratory signals

Songs were recorded at room temperature (24 ± 3° C) with a condenser microphone (4133, Brüel & Kjaer, www.bksv.com) and a Brüel & Kjaer measuring amplifier (2610). A high pass filter of 300 Hz, 1 kHz, or 2 kHz was used. During recording of wing movements (see below), an electret microphone (Conrad Electronics, www.conrad.com) was used. Recorded songs were stored on digital tape (Pioneer D-C88, www.pioneer.ip) with a sample rate of 96 kHz. For analysis of temporal structure and production of figures, songs were digitized with an AD-converter (Analog Devices (www.analog.com) and TurboLab 4 (www.turbolab.de), rate 50 kHz) and viewed and analyzed with the program Neurolab (Hedwig and Knepper 1992).

Vibratory signals were recorded with a vibration detector (Brüel & Kjaer 4369) that was connected to a 50 g metal cylinder (Brüel & Kjaer) and positioned without further fixing on the base, frame, or top of the cage in which the insect was singing, usually within 20 cm from the insect. The detector was connected to an amplifier (Brüel & Kjaer Nexus conditioning amplifier), and vibratory signals were stored together with acoustic signals on digital tape. Analysis was as described for sound with the program Neurolab.

**Video 2.**
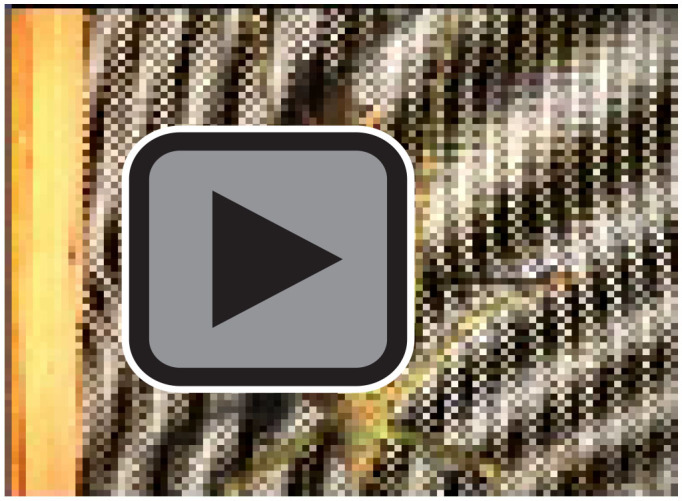
Recording of a *Xerophyllopteryx fumosa* male in a cage after removal of the hind wings. The bright spot on the left tegmen represents a piece of reflecting foil for recording wing movements. Before singing, the fore wings sometimes were held opened, and a cleft shows the internal cavity. The cleft may be much broader then seen in the video. Download video

### Recording of movements of fore wings and abdomen

Wing movements were recorded using position sensitive detectors coupled with an optical system (Helversen and Elsner 1977). On the left fore wing, a piece of reflecting foil was attached in the vicinity of the mirror using Fixogum (Marabu, www.marabu.com) (see Videos 1 and 2). Movement of the abdomen was measured as the direct reflection of the posterior abdomen, therefore not necessarily representing movement of an exactly defined point. The data were either stored on digital tape (Pioneer DC-88) or directly digitized using a National Instrument AD-card (NI PCI 6251) and Lab View 9 (www.ni.com). Data were visualized and figures produced using either the Neurolab program or Diadem 9 or 10 (National Instruments).

### Frequency analysis

The frequency spectra of songs were analyzed from singing insects directly using a Hewlett Packard Spectrum Analyzer (HP 3567A, www.hp.com), a condenser microphone (1/2″ 4133 or 1/4″ 4135, Brüel & Kjaer), and a Brüel & Kjaer preamplifier (Type 5935). It was attempted to have the microphone in a position approximately 10 cm dorsal of the singer, but because the insects were sitting in cages (wooden frame, metal mesh; front “door” open) when singing, the exact position of the microphone relative to the insect varied between recordings. For calculating peaks of frequency spectra, some measurements were taken from original data (accuracy 0.128 kHz, <0.1 dB), and other data were measured from stored power spectra (accuracy ca. 0.1 kHz and 0.1 dB). For each spectrum, more than 10 verses were averaged, and recording position varied slightly. For measuring peak amplitudes, the distance between the microphone and the insect was more rigidly controlled (10 ± 1 cm).

### Behavioral observations

Wing movements of *X. fumosa* were recorded with position detectors and observed using a digital camcorder (Canon MV10, www.canon.com) and digital photos (Canon Powershot G3). Special focus was placed on the positioning of the hind wings and the extension of the wings when opening and closing before and after treatment. The behavior of *N. reticulatus* (singing, tremulation, potential interaction with a female close by) in a cage (about 20 cm × 20 cm × 30 cm) under red light conditions was observed. Mirrors were removed from the tegmina and wings were removed after anesthetizing the insects with CO^2^ for 3 minutes. Immediately after the removals, the insects were returned to their cages. Singing may have occurred within one hour after the procedure, but sometimes it only occurred on the following day. Even untreated animals did not sing every day.

### Terminology of acoustic signals

The nomenclature of acoustic signals is quite diverse in different publications. Here, terms often used in cricket and many bush-cricket song descriptions are used. The term “phonatome” (e.g. Walker and Dew 1972) denominates all sound produced during one complete movement cycle (opening and closing) of the tegmina. In the majority of species, sound is produced only during the closing movement, and this sound most often is referred to as a “syllable.” One may discriminate between an “opening syllable” and a “closing syllable.” Unfortunately, when fore wing movements are not recorded, a clear assignment of song elements and fore wing movements is not possible. A pair of two syllables, one softer and one louder, does not necessarily mean that they together represent one opening and closing of the fore wings (see *N. reticulatus* song). A group of syllables or phonatomes typically presented in a sequence will be called a verse. A group of verses is called a song.

## Results

### Nesonotus reticulatus

**Figure 2.**
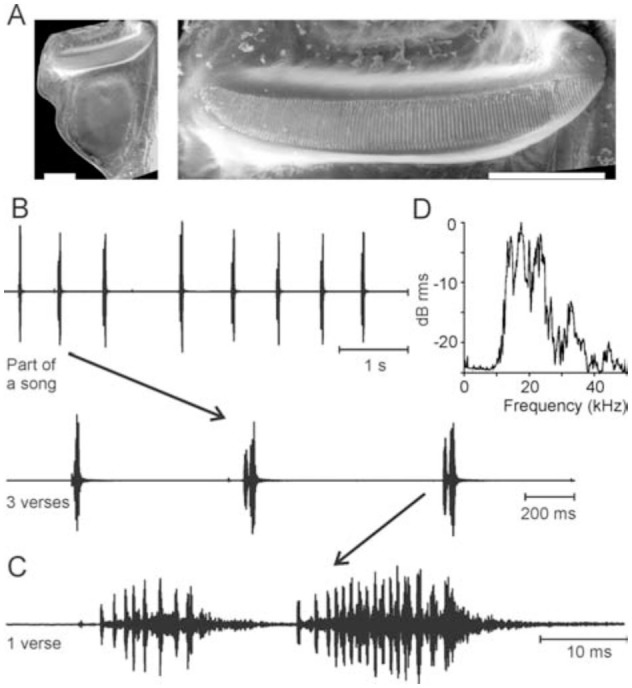
Song and wing of male *Nesonotus reticulatus* (laboratory reared F1-generation). **A:** Scanning electron microscopy pictures of the left wing (underside) and the stridulatory file on the left wing. Bars: 1 mm. **B:** Part of a song recorded at 24° C. The extension shows three verses containing one or two syllables. **C:** One typical two-syllable verse. **D:** Song spectrum. The largest peak was at 17.5 kHz. High quality figures are available online.

**Figure 3.**
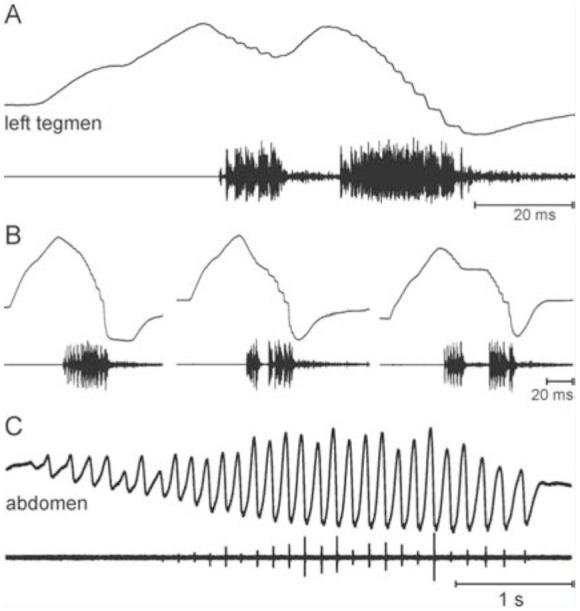
Sound production with tegmina (**A, B**) and tremulation with abdomen (**C**) in a male *Nesonotus reticulatus*. **A:** One typical verse produced by a partial and a full wing closure. **B:** Three stridulation movements of the same individual as in **A** show a gradual transition from a verse with just one syllable to a verse with two syllables. A complete closure compares to about 3.5 mm movement in the stridulatory area. Upper traces show movement, lower traces show sound respectively vibration produced by the movement. Up in the movement traces means wing opening (**A, B**) and lifting the abdomen (**C**). High quality figures are available online.

The singing apparatus of *N. reticulatus* was well-developed, the stridulatory area of the left fore wing (tegmen) not membranous, with a network of secondary veinlets (Figure 2A). The stridulatory file had around 121–135 lamellar teeth (n = 3) that at the edges may be hard to distinguish from small knob-like emergences. The right tegmen bore a large mirror (Figure 2A) but no stridulatory file. The song of *N. reticulatus* (Figure 2B, C) was relatively simple, as a unit of two (rarely one) syllables was presented in long lasting repetitions. As shown in Figure 3, these did not represent a phonatome, and therefore are called a verse. Verse duration was 30.6 ± 1.0 ms (n = 142, male 1) and 49.7 ± 3.7 ms (n = 160; male 2, both at 22–23°C). A third male at 26° C produced verses of 33.1 ± 4.2 ms (n = 56). The verses occured at irregular intervals of several hundred ms to more than a second (mean 646.6 ± 182.2 ms, N = 7 males, n = 1227 verses; range of means 447.3 ms to 960.2 ms). The sound was emitted with a frequency spectrum ranging from 10 to about 40 kHz, with the main energy between 15 and 25 kHz (Figure 2D). The two syllables were produced during the closing movement of the fore wings (Figure 3A), with the first, shorter syllable resulting from an incomplete closure. A syllable comprises a number of pulses, which are produced during accelerated closing movements and may be interrupted by more or less clearly expressed pauses, when the fore wing movement is slowed down. Often, however, sound is also produced during the slower movement and a pulsed structure is not very obvious. Figure 3B shows recordings of movements of one individual at the beginning of a singing period, which demonstrate that there can be transitions between a verse comprising just one syllable (by just one continuous or only briefly interrupted closure of the tegmina) and those comprising two syllables (then typically, but not necessarily, a second opening of the fore wings is interspersed). However, even in the one syllableverse one can typically see two different phases by different pulse repetitions rates.

**Figure 4.**
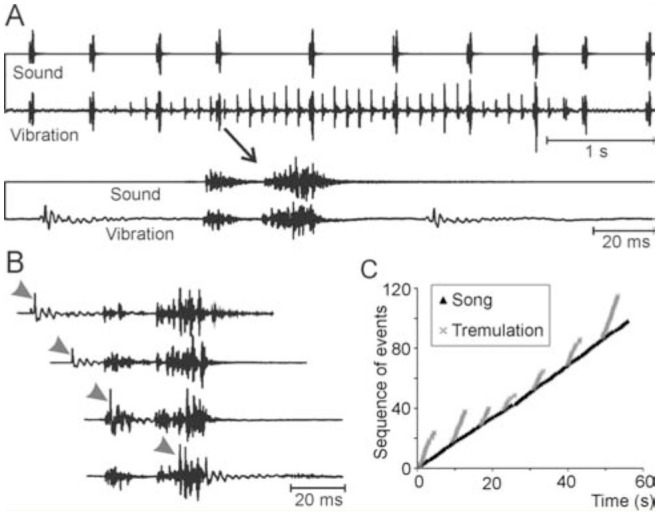
Song and vibratory signal from a male *Nesonotus reticulatus*. **A, B:** The upper trace in each recording is from a microphone (“sound”), the lower from a vibration detector attached to the ground close to the male (“vibration”). The vibratory signal was elicited by tremulation with the abdomen. **A:** Common occurrence of singing and tremulation. **B:** Independence of singing and tremulation. The grey arrowhead points at the start of the tremulation signal. The acoustic signals are shown aligned to each other. All signals are taken from one longer recording. **C:** Temporal relation of a longer sequence of singing and tremulation. While the singing occured at a relatively constant rate of 1.75 Hz, tremulation sequences were faster, shorter, and occurred with intervals of about 5 sec. High quality figures are available online.

In addition to singing, males produced a tremulation with their large and heavy abdomens (Figure 3C). This signal occurred independently of but also during singing. (Figure 4). The single pulses occurred with about 5–10 Hz repetition rate (mean interval 156.0 ± 34.4 ms, N = 7 males, n = 1550 intervals). Usually, 17–46 pulses were grouped (mean 30.6 ± 8.2; N = 7 males, n = 56 tremulations) and presented with slowly increasing and finally decreasing amplitude (Figure 3B, 4A). The vibrations elicited by the tremulation were below 1 kHz, but all were recorded on artificial substrate, only. Tremulation and singing sometimes appeared to be phase coupled. In general, however, they were not (Figure 4B, C). Both were produced quite regularly, but with their independent rhythms (Figure 4C). The frequency of occurrence of tremulation seemed to strongly increase after contact with females. Nearly uninterrupted series of hundreds of tremulation pulses in sequence occurred. The amplitude, though, appeared to be lower in such cases. Singing was strongly reduced or completely abolished in such situations. In one case, a female was observed tremulating softly and shortly as well. Additionally, tremulation appeared to serve as a signal in direct contact with other males, as it was produced at shorter intervals with two males alternating. This finding is supported by the observation that tremulation by males was easily elicited as a response to artificial drumming on the cages. On the other hand, several males sang in close distance for an hour or longer without tremulating.

### Karukerana aguilari

**Figure 5.**
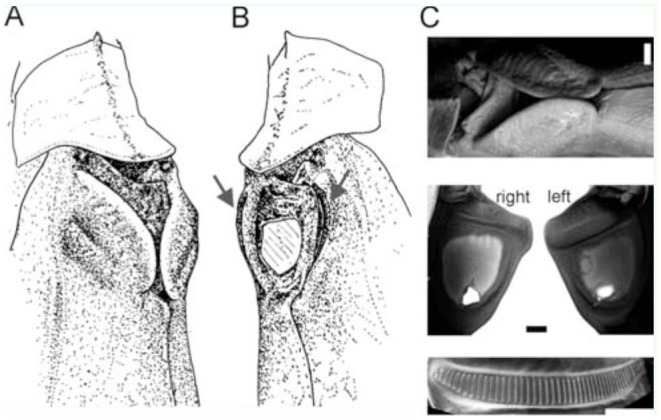
Wing morphology in *Karukerana aguilari*; anterior is up. **A:** Dorsal view of the pronotum and the large fleshy flaps extruding from the anterior fore wings. **B:** Dorsal view after removal of the flaps (grey arrows point at the former insertion site). Now the membranous mirror on the left tegmen can be seen. **C:** Scanning electronic picture of the extrusions, the tegmina, and the stridulatory file on the ventral side of the left tegmen. The holes in the two mirrors indicate that both are membranous, even though the left mirror may be thicker than the right mirror. Bars in **C:** 1 mm. High quality figures are available online.

The sound producing apparatus of *K. aguilari* was nearly completely covered by two extrusions of the tegmina and by the pronotum's hind margin (Figure 5A, C). Both fore wings contained large, thin, membranous mirrors (Figure 5C). Nevertheless, a clear fore wing asymmetry existed, with a stridulatory file only on the ventral side of the left fore wing, and a plectrum only on the right fore wing. The file had 43–45 broad teeth (n = 4).

**Figure 6.**
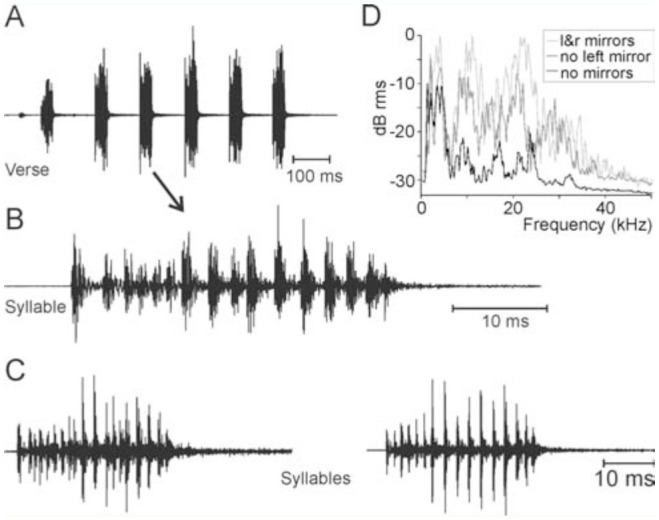
Verse and syllables of a male *Karukerana aguilari* at 22° C. **A:** One complete verse. **B:** One syllable (likely one phonatome). **C:** Two syllables of the same male, recorded at two different positions above the male (26° C). **D:** Song spectra before and following certain manipulations (see text). For better comparison, the spectrum for “no left mirror” starts at -5 dB, and the spectrum for “no mirrors” starts at -10 dB. Before lesions, the largest peaks were at 4.1, 11.1, and 21.6 kHz. After removal of the folds and of the left mirror they were at 2.2, 4.1, 10.2, and 20.7 kHz. Without folds and any mirror they were at 1.4 and 3.5 kHz. High quality figures are available online.

A song consisted of irregularly spaced verses (pauses 7.2 ± 0.4 sec, n = 27, and 9.8 ± 3.1 sec, n = 14, two days of recording), each consisting of 4–6 syllables, likely produced during one closing movement of the tegmina (Figure 6A–C). The syllables were produced with a period of 126.3 ± 11.5 ms (n = 29). A syllable was clearly structured into pulses (10–20) of about 2 ms duration (Figure 6B, C). The stridulation was a rattling sound, with a broad spectrum containing maxima between 2 and 22 kHz (Figure 6D). The song was extremely loud, as 125–128 dB SPL peaks were measured at a distance of 10 cm dorsally above insect.

To assess a putative influence of the folds over the song-producing organ on sound production, the folds were removed. Removing both folds did not change the song in any obvious way. Neither sound intensity nor spectral composition were altered. The function of the folds may be found in the fact that in contrast to the majority of Tettigoniids, in *K. aguilari* both forewings contain thin, membranous mirrors (Figure 5C). Destroying the left mirror membrane, but leaving the frame intact, reduced the high frequency peaks slightly (Figure 6D), but decreased intensity (peak dB SPL) by only about 3 dB. Additionally destroying the right mirror immediately reduced the song intensity by about 10 dB to maximum values of 112 dB SPL peak. At the same time, the high frequency energy above 7 kHz was strongly reduced (Figure 6D). When excising the right mirror, a tiny hole in the membrane was detected. It is not clear whether it was created during excision of the left mirror, or whether it was there before. Nevertheless, it apparently did not dramatically affect sound production, because completely excising the mirror (bearing the hole) had the most dramatic effect of all manipulations. As for sound amplitudes, peak levels, even when recorded at the same position, varied by 5 dB, and measuring at the same distance but at different positions relative to the male may bring another 7 dB variation. Because the songs were recorded on different days (following a manipulation, the animal did not sing any more on that day) with different positions of the animal in a relatively large cage (25 cm × 20 cm × 20 cm), absolute constancy of recordings cannot be expected. The peak amplitudes presented here, however, were the highest values recorded at 10 cm distance at various positions more or less dorsally above the animal.

### Xerophyllopteryx fumosa

**Figure 7.**
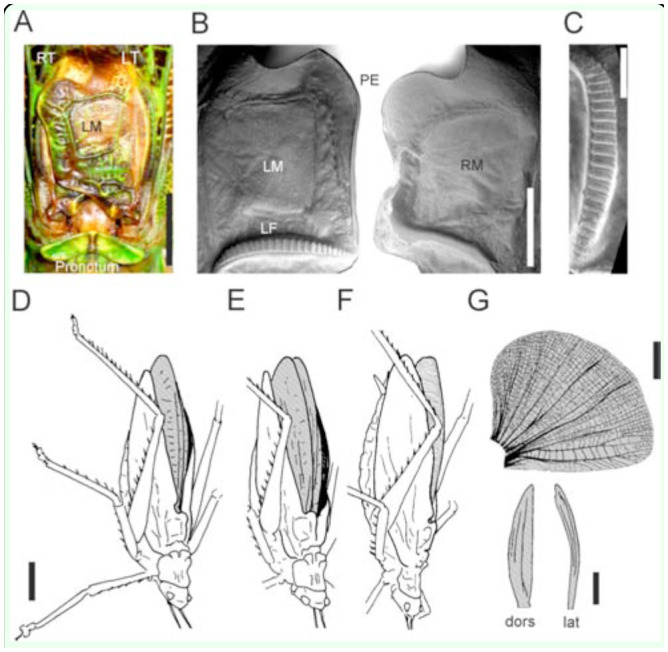
Wings and song postures of a male *Xerophyllopteryx fumosa*. Since this species typically sung with the head down, all figures are oriented in this way. **A–C:** Photos and scanning electron microscopy pictures of the male stridulatory apparatus. **A:** Dorsal view on the stridulatory region in an intact insect with the left tegmen (LT) with a thick mirror (LM) covering the right tegmen (RT). **B:** Ventral side of the left tegmen with stridulatory file and of the right tegmen carrying the membranous mirror (RM). PE = posterior edge. **C:** Enlarged view on the stridulatory file. **D:** When a male was ready to sing, the tegmina were slightly opened and lowered, while the hind wings (dark) were raised. **E:** During singing, the fore wings were opened in the lowered position, and the hind wings were further opened. **F:** After removal of the hind wings, a male ready to sing opened the fore wings, similar to the intact insects (the grey area shows the inside of the fore wing). **G:** Extended (upper) and folded (lower) hind wings from a dorsal and lateral view. Bars in **A:** 5 mm, in **B:** 2 mm, in **C:** 1 mm. Bars in **D**–**G**: 10 mm*.* High quality figures are available online.

The sound producing apparatus of *X. fumosa* was characterized by the distal border of the anal field delineating a lobe (angulose in the left fore wing, rounded in the right one) followed by a concavity (Figure 7A, B). Only the mirror of the right tegmen was membranous, and only the ventral side of the left tegmen had a strongly-curved stridulatory file with 26–30 broad teeth (n = 3) (Figure 7B, C). During singing, the tegmina, which in most long-winged bush-cricket species are lifted to some degree, were held in a very unusual position, with the tips pointing downwards (Figure 7D– F). This led to a long, open cleft between the tegmina. In this cleft, the partially raised and slightly opened hind wings could be seen (Figure 7D, E; Video 1). Because songs were always produced in this position, the hind wings might influence sound emission.

**Figure 8.**
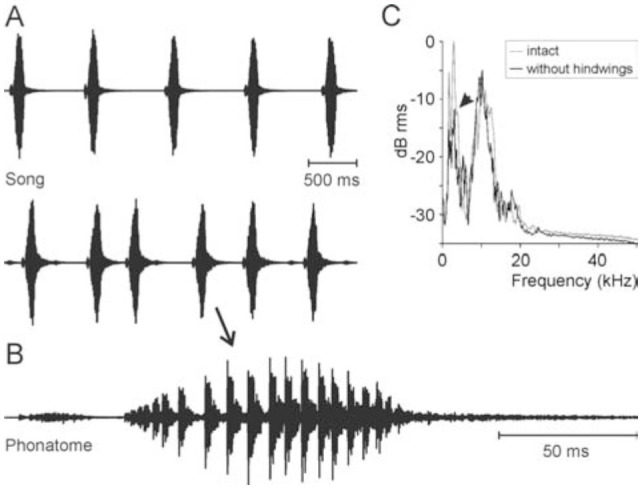
Songs of a male *Xerophyllopteryx fumosa* recorded at 26° C. **A:** Portions of two songs with differing rhythm. **B:** One phonatome. **C:** Song spectrum. For easier comparison, the spectrum “without hindwings” starts at -5 dB. The largest peak was at 2.9 kHz before manipulation, and at 10.2 kHz after removal of the hind wings. In comparison to the peak at 10 kHz, the peak around 3 kHz was clearly reduced after removal of the hind wings (see arrow). High quality figures are available online.

**Figure 9.**
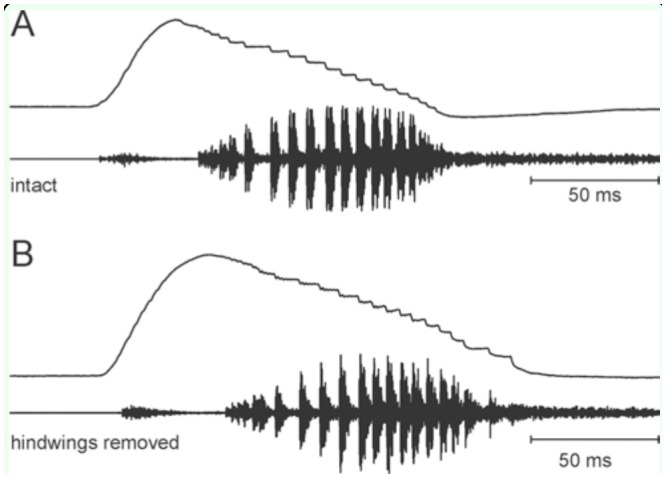
Songs of a male *Xerophyllopteryx fumosa* before (**A**) and after (**B**) removing the hind wings. The upper trace in A and **B** shows the wing movement (up means opening), and the lower trace shows the sound produced. **A** complete closure of the tegmina compares to about 4 to 5 mm movement of the stridulatory region. High quality figures are available online.

The intense song of *X. fumosa* had a maximum peak around 120 dB SPL at 10 cm distance. A song comprised more than 500 phonatomes (Figure 8A, B, 9A). The song spectrum had two distinct peaks, one between 1.7 and 3 kHz, and a second between 10 and 13 kHz (Figure 8C). A phonatome comprised a very soft sound produced during the opening of the fore wings (Figure 9A) and a dominant syllable comprising about 20 very distinct pulses, which were produced during a step-wise closing movement of the fore wings (duration: one individual 103.4 ± 12.3 ms at around 26° C, n = 577). Often, two phonatomes were produced with a shorter interval (e.g., 365.9 ± 28.9 ms, n = 109) than the following one (612.1 ± 93.0 ms, n = 450; see Figures 8A, 9A, B), giving the impression that a long song is divided into many verses of two phonatomes. However, this verse-like pattern does not necessarily exist (Figure 8A).

**Figure 10.**
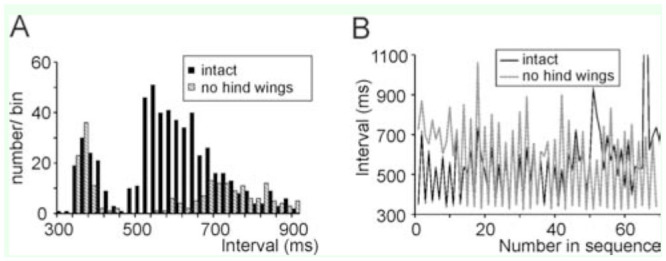
Verse-like rhythm of the *Xerophyllopteryx fumosa* song. In **A**, the intervals between phonatomes of one male are shown before and after removal of the hind wings (n = 572 before, and n = 211 after removal). A bimodal distribution indicates the existence of two rhythms, the second one potentially being slowed down following the removal of the hind wings. In **B**, parts of two songs of the same male as in **A** before and after removal of the hind wings show that in both situations there was a tendency of producing verses (a longer interval was followed by a shorter one). High quality figures are available online.

The hind wings had a relatively strong structure and were brownish in color (Figure 7G). In the anterior region, particularly in the radial field and to a lesser extent between the radius and media, veins and transverse veinlets were thickened, determining a relatively strong and ventral bending of the wing in the folded position (Figure 7G). After removal of the hind wings, the tegmina often were still opened before singing, revealing a large, concave base space formed by the curved tegmina and dorsal bending of the abdomen. Directly after a closing syllable, however, the wings usually were completely closed (Video 2). The song pattern was largely unchanged (Figure 9; duration of the closing movement was 97.9 ± 4.4 ms at around 24° C, n = 213). The verse-like structure still occurred, but the inter-verse interval was on average longer than in the intact situation (Mann-Whitney U-Test, n = 572 intact, 211 lesioned, *p* = 0.0027; Figure 10). The song intensity (measured in peak SPL at 10 cm distance dorsally of the intact insect), on the other hand, was reduced from 114–118 dB to 108–113 dB SPL peak (Mann-Whitney U-Test, *p* = 0.0001; n = 10-11). The clearest change was seen in the song spectrum (consistent in two individuals, Figure 8C). The intensity of the low-frequency peak was typically higher than that of the high-frequency peak (median difference high- minus low-frequency peak: 7.1 and 7.7 dB in two individuals, n = 5 and 6) when wings were intact, but the low-freqency intensity was in one individual clearly lower (median difference high- minus low-frequency peak: 6.1 dB, n = 5; 2-tailed Mann-Whitney U-Test: *p* < 0.01) after removal of the wings, and in another individual the median difference was around 0, but still significantly different from the situation with hind wings (median difference low-minus high-frequency peak: 0.3 dB, n = 5*, p* = 0.012). At the same time, the average frequency of the high-frequency peak was significantly lowered in both specimens by about 1 kHz (two-tailed t-test, *p* < 0.01 in one insect, *p* = 0.04 in the other; n = 5-6 in both), while the low-frequency peak was not significantly different. As the spectrum in Figure 8C shows, however, there were always two to three peaks in the low frequency-range and in the high-frequency range, and the differences between these peaks mostly were small.

## Discussion

*Nesonotus reticulatus* is distributed in the Southeast Carribean. It is known since the late 18^th^ century and was re-described by Bonfils (1966). It is a large species, brownish in color, and shows net-like structures on the wings. It lives from wet to more dry areas, from 50 to 500 m a.s.l. in altitude, between 1 and 5 m above ground, and also in secondary forest and garden areas. Its song was a rather short, high-pitched verse with a reasonable amount of energy in the audible range. The song can be produced for hours, with one to two verses per second. Most notably, however, was an additional concomitant signal, which was a tremulation of the heavy abdomen producing a vibratory pulse with every contact of the abdomen with the ground. This signal was obviously directed to both males and females. It was increased in frequency when in contact with females, while singing may have been reduced, but it was also produced in response to tremulations of other males, a behavior that easily was elicited by tapping against the sub-stratum the males sat on. In general, vibrations are also elicited by a bush-cricket song itself (Keuper et al. 1985, see also Figure 3A), and this vibration may increase attractiveness of an acoustic signal (e.g., Latimer and Schatral 1983). When singing species come into close contact, they may stop singing and instead start some kind of vibration by shivering their whole body (e.g., Stiedl and Kalmring 1989; Morris et al. 1994). Some bush-crickets, like *Myophyllum speciosum*, use differing tremulation signals when approaching a potential partner, both when within 1 m on the same substrate or when being in close proximity to a mate (Morris et al. 1994). Tremulation may serve as a means for short distance communication on a “private channel” not detectable by relevant predators such as bats (Römer et al. 2010). It still may be detectable, though, to other predators like spiders (e.g., Barth 2002; Virant-Doberlet et al. 2011). In *N. reticulatus,* however, the tremulation signal was a regular event occurring during as well as without singing. Such tremulation has been described for various acoustic and non-acoustic insects (e.g., Ewing 1989; Drosopoulos and Claridge 2006) and also for other Pseudophyllinae, in which this seems to be a widespread means of communication (Belwood 1990; Morris et al. 1994; Römer et al. 2010). The tremulations of *N. reticulatus* are similar to the “final vibration trains” described for the Pseudophylline *Choeroparnops gigliotosi* (Morris et al. 1994), but lack the variety of other forms observed in this species. To our knowledge, *N. reticulatus* is the only species where simultaneous calling song and tremulation occur. In *Ephippiger perforants,* a song is produced while tremulating, but it is structurally distinct from the calling song (Ragge and Reynolds 1998).

The role of the tremulation may be to produce short-range signals undetectable for acoustically hunting predators, as has been hypothesized for a Pseudophylline species from Panama, *Docidocercus gigliotosi*, which suffers from considerable pressure by bats (Römer et al. 2010). In accordance with this, *D. gigliotosi*, like *N. reticulatus*, produces a short acoustic signal with long pauses. *D. gigliotosi* seemed to trade the amount of tremulation (which is more expensive than singing) against the amount of singing depending on the conditions, especially in nocturnal light levels (Römer et al. 2010). It is unkown whether this is the case in *N. reticulatus* as well. In various bush-crickets, it has been observed that a female will reply with a tremulation when a male uses a vibratory signal (Belwood 1990), but this behaviour was only seen once in *N. reticulatus.*

*Karukerana aguilari* is endemic to Guadeloupe. It is a large, leaf-like species restricted to lowland forests and mountain rainforests. In low altitudes (from 100 m to 900 m a.s.l.) it lives on the canopy, while in higher altitudes it is seen on the upper leafs of *Phylodendron giganteum.* Males usually call several meters apart. Except for the first description by Bonfils (1965), no data are available. The call resembled that of the related species *Pterophylla camellifolia*, with its broad spectrum extending to low frequencies and by its gross temporal structure (Shaw 1968). We first hypothetized that the prominent and unusual folds, extruding from the tegmina and covering the area with the stridulatory structure, had a function in sound radiation. While this is true in other species, such as an undescribed Agraeciini (Conocephalinae; Morris and Mason 1995), where a protrusion of the pronotum covers the shortened wings and creates a specific space, removal of the two folds did not render any effect on sound intensity or sound spectrum. Therefore, these folds most likely have a protective function for the delicate structures used for stridulation. Unlike in the vast majority of tettigoniids, both tegmina of *K. aguilari* had similarly-sized membranous mirrors. Therefore, the usual protection of the one membranous mirror on the right tegmen by the thickened area of the left tegmen was missing here, as the left tegmen also had a membranous mirror. The protection instead may be provided by the folds. However, well-developed mirrors in the left wing also occur in Pseudophylline species without such folds, e.g., in other Pterophyllini (Beier 1960) such as *Pterophylla*, but also in other tribes (e.g. Montealegre and Morris 1999). In the Pseudophyllinae tribe Aspidonotini, where developed mirrors in the left wing also occur (Hugel, unpublished), the stridulatory apparatus is entirely protected by a large extension of the pronotum. Nevertheless, it must be assumed that developing a fully-formed membranous mirror on the left wing is some kind of re-evolution, because fossil species of Tettigoniids (but not Haglids) and the majority of extant species have asymmetrical wings (Rust et al. 1999; Gu et al. 2012).

The results might indicate that both mirrors contribute similarly to sound production, even though the left mirror might produce stronger high-frequency peaks. The 3 dB decrease in sound intensity after destroying one mirror would point to the existence of two independent sound sources. At the same time, the variation in measured sound show that singular observations can be unreliable and make a repetition of this experiment in a number of individuals necessary. The left and right wing might also acoustically interact in some other way, e.g., as two coupled oscillators driven by the same stridulatory movement, and the vibration of the left mirror itself might not be of major importance. A coupling between the left and right mirror, however, is mostly seen in pure tone singers utilizing resonances of both wings, as has been demonstrated in crickets (Montealegre et al. 2011). Moreover, it would have to be demonstrated that the left mirror actually exhibits relevant vibration, because in several species of Tettigoniids with membranous left and right mirrors, the vibration of the left mirror was considerably less than that of the right mirror (Fernando Montealegre-Z, personal communication). Destruction of both mirrors of *K. aguilari* mostly eliminated higher frequency peaks, while peaks below 5 kHz were still prominent, and the sound produced was still around 110 dB SPL peak, indicating a very effective sound radiation at low frequencies by the remaining wing structures, e.g., the mirror frame and other structures surrounding the mirror, while the thin membrane(s) might be effective amplifiers for higher frequencies only (see Morris and Pipher 1967; Bailey 1970; Montealegre and Mason 2005). Also, a left wing with a thick mirror area might most effectively radiate sound at frequencies below 10 kHz (Montealegre and Postles 2010). It should be mentioned that symmetry of the tegmina does not mean, neither in *K. aguilari* nor in *X. fumosa,* that the stridulatory file and scraper are similarly expressed on both wings. Only the left wing contained a well-developed file in both species, with relatively few teeth (30 to 40; see extensive data in Montealegre and Morris 1999).

*Xerophyllopteryx fumosa* is a large species with a bark-like appearance. It lives in wet areas from 100 to 800 m a.s.l. in forests with large, old trees. During the night, *X. fumosa* can be seen on the bark of trees, often in aggregations of 5 to 10 specimens on the same shaft. It produced a rather simple song in the audio range. The dominant peak below 4 kHz makes it sound like a frog call. Two unusual morphological features seemed to be involved in sound production. The complex shape of the stridulatory region (Figure 7) probably allows a closing movement with song production despite the low held tegmina. This tegminal position leaves room for a cavity, which was formed by lowered tegmina, lifted and slightly opened hind wings, and the curved abdomen. This cavity becomes most obvious after removing the hind wings. When the hind wings were removed, the song spectrum had a reduced low-frequency peak, indicating that the hind wings help in radiating the low frequencies. This might be due to a resonating property of the cavity formed by the tegmina, wings, and abdomen (e.g., Morris and Mason (1995) or Bennet-Clark (1999), discussing resonating properties of cicada abdomen). The specific dimensions of the cavity, however, were hard to judge. If it is assumed that the wings are as flat as the tegmina (which they are not, see Figure 7), air in the large cavity likely resonates at rather high frequencies, unless the cavity opening is very small. Following considerations made by Bailey and Broughton (1970), a cavity with a length around 45 mm might allow frequencies around 3.7 kHz to resonate in the air column between the tegmina, wings, and abdomen. The low frequencies in the song, however, were lower, and the dominant frequencies in the spectrum itself did not change much with the manipulation. Only the relative amplification of frequencies changed. A reasonable estimation of the internal space and opening of the cavity was unable to be made; therefore, the properties of this cavity remain speculation. The high-frequency peak in the insects with no parts removed was somewhere between 11 and 12.5 kHz (Figure 8C), which fits the correlation between file length (here around 4.5 mm) and carrier frequency found for a large sample of bush-cricket species (Montealegre 2009; Gu et al. 2012).

It is concluded that the hind wings contributed considerably to increasing sound radiation in the low-frequency range by a mechanism that is yet to be identified. This strengthening of low frequencies will directly increase the distance over which the song can be heard, which may be quite important for diurnal animals in the rain forest. It is not known if a similar role of the hind wings has been described for other ensiferan species.

## References

[bibr01] Bailey WJ. (1970). The mechanics of stridulation in bush crickets (Tettigonoidea, Orthoptera). I. The tegminal generator.. *Journal of Experimental Biology*.

[bibr02] Bailey WJ, Broughton WB. (1970). The mechanics of stridulation in bush crickets (Tettigonoidea, Orthoptera). II. Conditions for resonance in the tegminal generator.. *Journal of Experimental Biology*.

[bibr03] Bailey WJ, Rentz DCF., Bailey WJ, Rentz DCF (1998). The Tettigoniidae - their diversity and importance in biological research.. *The Tettigoniidae: Biology, Systematics and Evolution*.

[bibr04] Barth FG. (2002). *A Spider's World: Senses and Behavior.*.

[bibr05] Beier M., Mertens R, Hennig W, Wermuth H (1960). Orthoptera, Tettigoniidae (Pseudophyllinae II).. *Das Tierreich*, issue 74..

[bibr06] Belwood JJ., Bailey WJ, Rentz DCF (1990). Anti-predator defences and ecology of neotropical katydids, especially the Pseudophyllinae.. *The Tettigoniidae: Biology, Systematics and Evolution*.

[bibr07] Bennet-Clark HC. (1999). Resonators in insect sound production: how insects produce loud pure-tone songs.. *Journal of Experimental Biology*.

[bibr08] Bonfils J. (1965). Contribution à l'étude des Orthopteroides des Antilles. I. *Karukerana aguilari* n. gen. n. sp.. *Bulletin de la Société Entomologique de France*.

[bibr09] Bonfils J. (1966). Contribution à l'étude des Orthoptéroïdes des Antilles, II.. *Bulletin de la Société entomologique de France*.

[bibr10] Drosopoulos S, Clardige MF. (2006). *Insect sounds and communication: physiology, behaviour, ecology and evolution.*.

[bibr11] Ewing AW. (1989). *Arthropod bioacoustics: neurobiology and behaviour.*.

[bibr12] Gu JJG, Montealegre-Z F, Robert D, Engel MS, Qiaod GX, Ren D. (2012). Wing stridulation in a Jurassic katydid (Insecta, Orthoptera) produced low-pitched musical calls to attract females.. *Proceedings of the National Academy of Sciences USA*.

[bibr13] Gwynne D. (2001). *Katydids and bush-crickets. Reproductive behaviour and evolution of the Tettigoniidae.*.

[bibr14] Hedwig B, Knepper M. (1992). Neurolab, a comprehensive program for the analysis of neurophysiological and behavioural data.. *Journal of Neuroscience Methods*.

[bibr15] Heller KG. (1988). *Bioakustik der europäischen Laubheuschrecken.*.

[bibr16] Heller KG. (1995). Acoustic signalling in palaeotropical bushcrickets (Orthoptera: Tettigonioidea: Pseudophyllidae): does predation pressure by eavesdropping enemies differ in the Palaeo- and Neotropics?. *Journal of Zoology*.

[bibr17] Helversen O von, Elsner N. (1977). The stridulatory movements of acridid grasshoppers recorded with an opto-electronic device.. *Journal of Comparative Physiology*.

[bibr18] Keuper A, Otto C, Latimer W, Schatral A., Kalmring K, Elsner N (1985). Airborne sound and vibration signals of bushcrickets and locusts; their importance for behavior in the biotope.. *Acoustic and vibrational communication in insects*.

[bibr19] Korsunovskaya OS. (2008). Acoustic Signals in Katydids (Orthoptera, Tettigonidae). Communication I.. *Entomological Review*.

[bibr20] Latimer W, Schatral A. (1983). The acoustic behaviour of the bushcricket *Tettigonia cantans*. I. Behavioural responses to sound and vibration.. *Behavioural Processes*.

[bibr21] Montealegre-Z F. (2009). Scale effects and constraints for sound production in katydids (Orthoptera: Tettigoniidae): correlated evolution between morphology and signal parameters.. *Journal of Evolutionary Biology*.

[bibr22] Montealegre-Z F, Jonsson T, Robert D. (2011). Sound radiation and wing mechanics in stridulating field crickets (Orthoptera: Gryllidae).. *Journal of Experimental Biology*.

[bibr23] Montealegre-Z F, Guerra PA, Morris GK. (2003). *Panoploscelis specularis* (Orthoptera: Tettigoniidae: Pseudophyllinae): extraordinary female sound generator, male description, male protest and calling signals.. *Journal of Orthoptera Research*.

[bibr24] Montealegre-Z F, Mason AC. (2005). The mechanics of sound production in *Panacanthus pallicornis* (Orthoptera : Tettigoniidae : Conocephalinae): the stridulatory motor patterns.. *Journal of Experimental Biology*.

[bibr25] Montealegre-Z F, Morris GK. (1999). Songs and systematics of some Tettigoniidae from Colombia and Ecuador. I. Pseudophyllinae (Orthoptera).. *Journal of Orthoptera Research*.

[bibr26] Montealegre-Z F, Postles M. (2010). Resonant sound production in *Copiphora gorgonensis* (Tettigoniidae: Copiphorini), an endemic species from *Parque Nacional Natural Gorgona*, Colombia.. *Journal of Orthoptera Research*.

[bibr27] Morris GK, Mason AC, Wall P, Belwood JJ. (1994). High ultrasonic and tremulation signals in neotropical katydids (Orthoptera, Tettigoniidae).. *Journal of Zoology (London)*.

[bibr28] Morris GK, Mason AC. (1995). Covert stridulation: novel sound generation by a South American katydid.. *Naturwissenschaften*.

[bibr29] Morris GK, Pipher RE. (1967). Tegminal amplifiers and spectrum consistencies in *Conocephalus nigropleurum* (Bruner), Tettgoniidae.. *Journal of Insect Physiology*.

[bibr30] Ragge DR, Reynolds WJ. (1998). *The songs of the grasshoppers and crickets of western Europe*..

[bibr31] Römer H, Lang A, Hartbauer M. (2010). The Signaller's Dilemma: A Cost-Benefit Analysis of Public and Private Communication.. *PLOS ONE*.

[bibr32] Rust J, Stumpner A, Gottwald J. (1999). Singing and hearing in an ancient bushcricket.. *Nature*.

[bibr33] Shaw KC. (1968). An analysis of the phonoresponse of males of the true katydid, *Pterophylla camellifolia* (Fabricius) (Orthoptera: Tettigoniidae).. *Behaviour*.

[bibr34] Stiedl O, Kalmring K. (1983). The importance of song and vibratory signals in the behaviour of the bushcricket *Ephippiger ephippiger* Fiebig (Orthoptera, Tettigoniidae): Taxis by females.. *Oecologia*.

[bibr35] Virant-Doberlet M, King RA, Polajnar J, Symondson WOC. (2011). Molecular diagnostics reveal spiders that exploit prey vibrational signals used in sexual communication.. *Molecular Ecology*.

[bibr36] Walker TJ, Dew D. (1972). Wing movements in calling katydids: fiddling finesse.. *Science*.

